# Minimally Invasive Coracoclavicular Ligament Reconstruction Using Semitendinosus Autograft in a Case of Cho's Type IIC Lateral End Clavicle Fracture With Torn Conoid and Trapezoid Ligaments

**DOI:** 10.7759/cureus.32761

**Published:** 2022-12-20

**Authors:** Shirsha Ray, Vinod Nair

**Affiliations:** 1 Orthopaedics, Dr. D. Y. Patil Medical College, Hospital & Research Centre, Pune, IND

**Keywords:** semitendinosus autograft, modified classification, orthopaedics, coracoclavicular ligament reconstruction, clavicle

## Abstract

Clavicle fractures are a common clinical problem that accounts for about 10% of all fractures. Cho's type II fractures compromise the integrity of the coracoclavicular ligament and are thus inherently unstable, necessitating a lengthy healing period and being associated with a high rate of nonunion or malunion. The lowering of these rates is largely dependent on restoring the stability of the distal clavicle.

In our case report, a 60-year-old male came to the OPD with complaints of pain over the right shoulder for two days following a fall with his arm in an adducted position. He also complained of an inability to abduct the right shoulder beyond 45 degrees. A plain radiograph was done, which was suggestive of a right lateral end clavicle fracture with increased coracoclavicular distance compared to the uninvolved shoulder.

He was taken up for surgery after routine laboratory investigations and pre-anaesthesia check-up and minimally invasive coracoclavicular ligament reconstruction was done using a semitendinosus autograft. Intra-operatively, both the conoid and trapezoid ligaments showed tears. A universal shoulder immobilizer was applied post-surgery and continued for six weeks. Pendular exercises of the shoulder were started as per tolerance, and the patient responded well to surgery.

## Introduction

Distal clavicle fractures comprise 10-30% of all clavicle fractures. Fifty percent of these fractures are displaced and when treated conservatively, can result in 10-44% of instances of symptomatic malunion or nonunion [1,2]. The Neer-Craig subtypes IIA, IIB, and V of displaced fractures often require surgical treatment [3,4]. The tiny "extralateral" type IIC, in which both of the coracoclavicular (CC) ligaments are detached from the medial fragment, was included in a modified classification system proposed by Cho et al. [5]. Because of this type's small size, traditional hardware may not be suitable, necessitating the use of alternative CC stabilisation methods [6].

In our case report, we have reconstructed the anatomy of a coracoclavicular ligament (both conoid and trapezoid) in a Cho's type IIC lateral end clavicle fracture using a semitendinosus autograft.

## Case presentation

Clinical history

**Figure 1:**
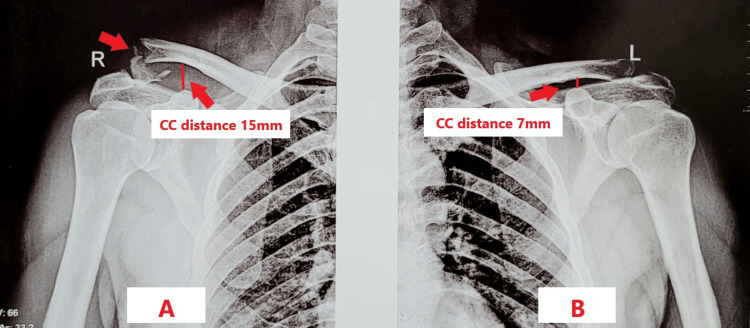
Pre-operative radiograph (A) Anteroposterior (AP) view of the right shoulder showing lateral end clavicle fracture and coracoclavicular (CC) distance of 15 mm. (B) AP view of the left shoulder (unaffected) showing a coracoclavicular distance of 7 mm.

A 60-year-old male came to the outpatient department (OPD) with complaints of pain and restriction of movements in the right shoulder for two days. He was apparently well two days prior to the hospital visit, when he slipped and fell with his arm in the adducted position, following which he developed pain over the right shoulder which was acute in onset, continuous in nature, aching in character, moderate in severity, aggravated by movement, and relieved by rest, immobilization and pain medication. A plain radiograph was done, showing lateral end clavicle fracture with increased coracoclavicular distance compared to the uninvolved shoulder as shown in Figure 1A, 1B.

On examination, there was tenderness over the acromioclavicular joint and restriction of movements of the right shoulder. Active forward flexion and abduction were possible up to 45 degrees.

He was taken up for surgery after routine laboratory investigations and pre-anaesthesia check-up.

Surgical procedure

Semitendinosus Graft Harvesting

**Figure 2:**
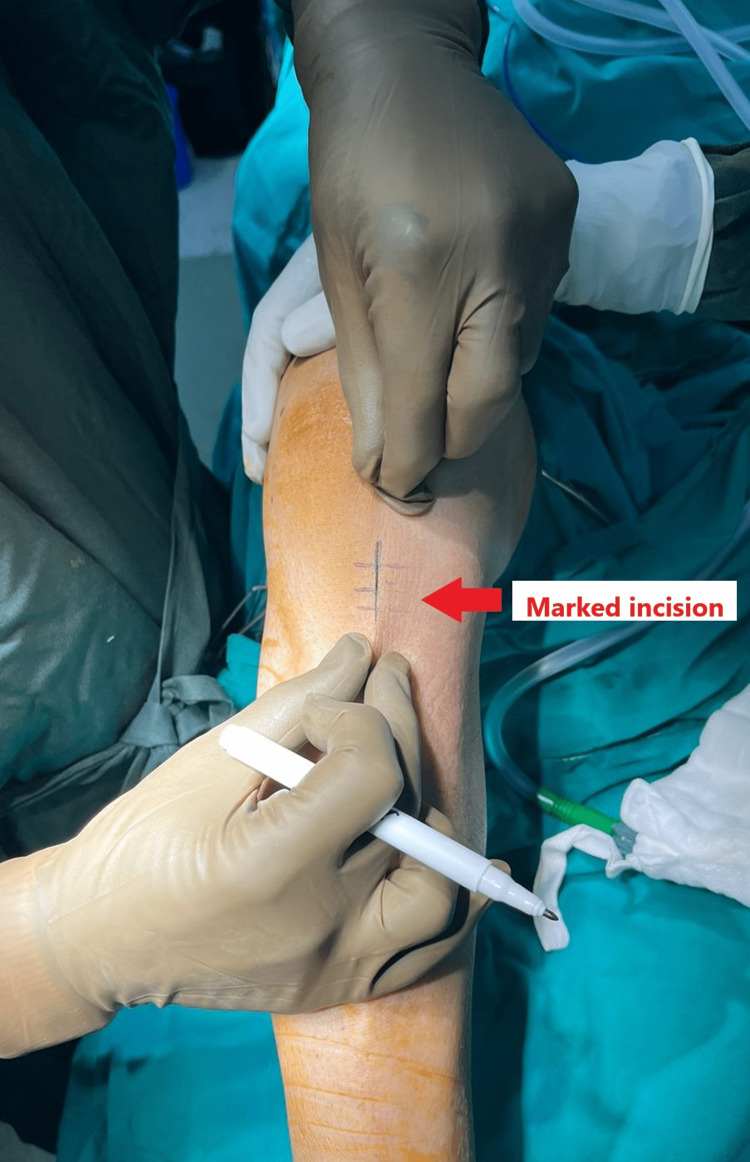
Incision for semitendinosus graft harvesting.

The procedure was done under general anaesthesia (GA) with the patient in the supine position. The right lower limb was exsanguinated, and the tourniquet was inflated to 280mm of Hg. In 90 degrees of knee flexion, the pes anserinus tendons were located manually below the skin. A 3-4 cm vertical incision was then made just 2 cm above the tendons and 2 cm medial to the anterior tibial tubercle, as shown in Figure 2.

**Figure 3:**
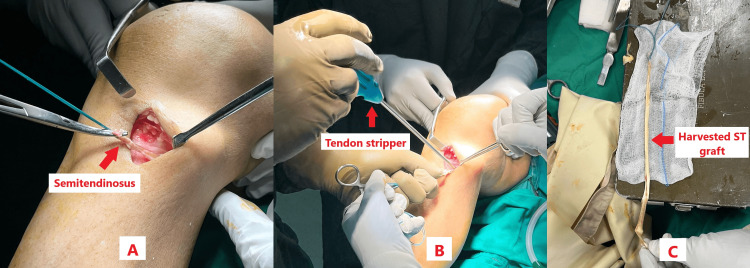
Semitendinosus graft harvesting. (A) Semitendinosus dissected. (B) Open stripper used to harvest semitendinosus. (C) Harvested graft of approximately 25 cm.

Using dissecting scissors, soft tissues were divided into the tiniest sections possible, up to the fascia superficialis. The two tendons were then precisely visible through the aponeurosis after the fascia was meticulously cleaned with a swab. Just above the tendons, a horizontal 3 cm incision was made to open the fascia. A Landager hook and probe were used to grasp the gracilis tendon. To separate the two tendons and guarantee that the semitendinosus was collected, the gracilis was positioned beneath the retractor. The same technique was used to alternate between grabbing the semitendinosus with a probe and a hook. The semitendinosus was then held with curved artery forceps, and an open tendon stripper was used to progressively remove the semitendinosus tendon to harvest a graft of approximately 25 cm in length as shown in Figure 3A-3C.

Coracoclavicular Ligament Reconstruction

**Figure 4:**
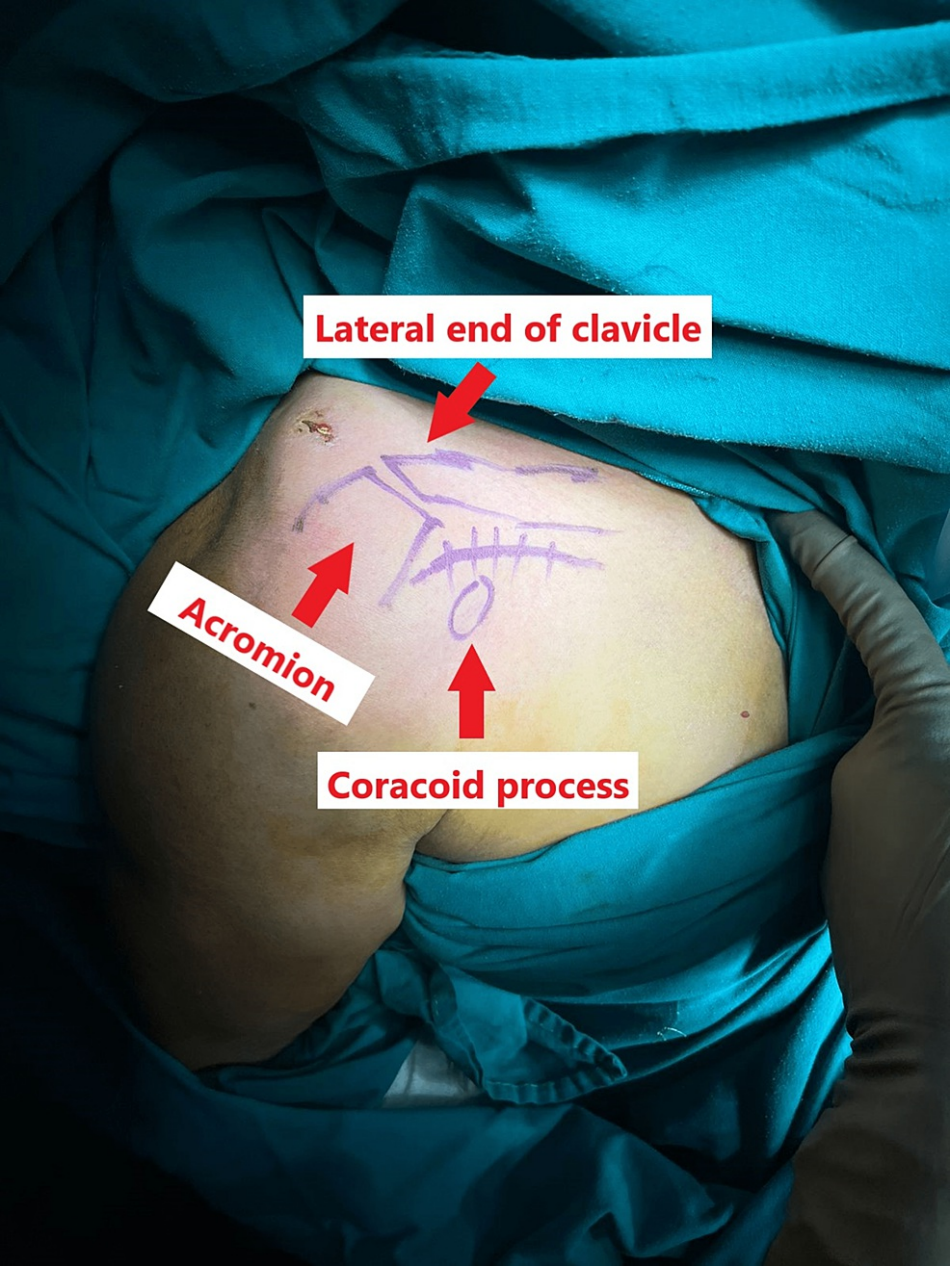
Bony landmarks and incision marked.

The patient was given a beach chair position with the image intensifier on the other side of the table. A 4-5 cm transverse arcuate incision centred over the coracoclavicular ligament was made after marking the acromion, lateral end of clavicle and coracoid, as shown in Figure 4.

**Figure 5:**
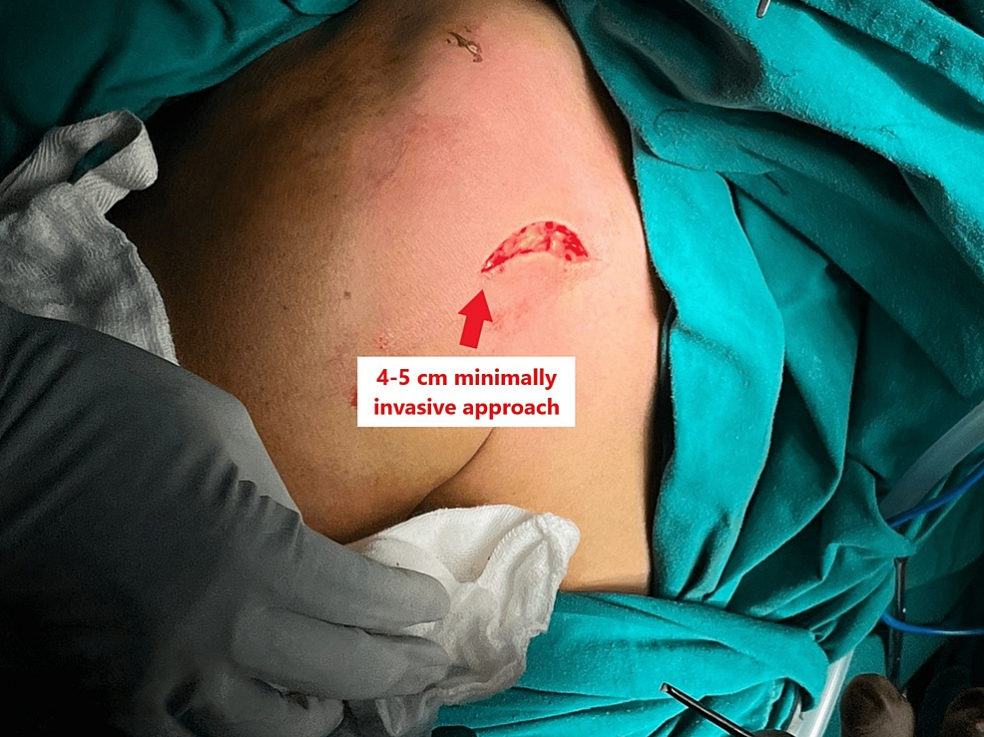
Minimally invasive approach to the coracoclavicular ligament.

This minimal approach, shown in Figure 5, allowed adequate exposure of the lateral end of clavicle and coracoid process.

**Figure 6:**
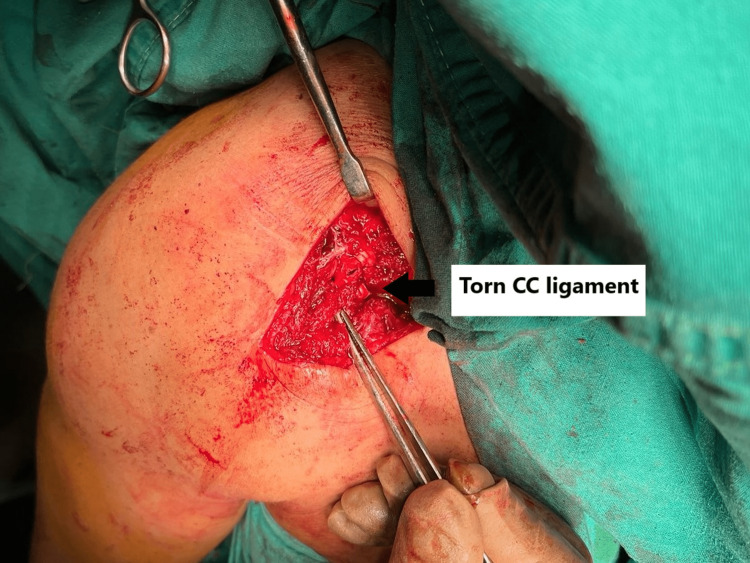
Torn conoid and trapezoid ligaments.

The lateral third of the clavicle and fracture, as well as the AC joint, were exposed by subperiosteally elevating a T-shaped flap raised from the deltotrapezial fascia. Blunt dissection through the anterior deltoid along the vertical limb of the T-flap was done to expose the coracoid. Both the conoid and trapezoid ligaments were found to be torn, as shown in Figure 6, deeming the fracture Type IIC according to Cho's modified classification.

**Figure 7:**
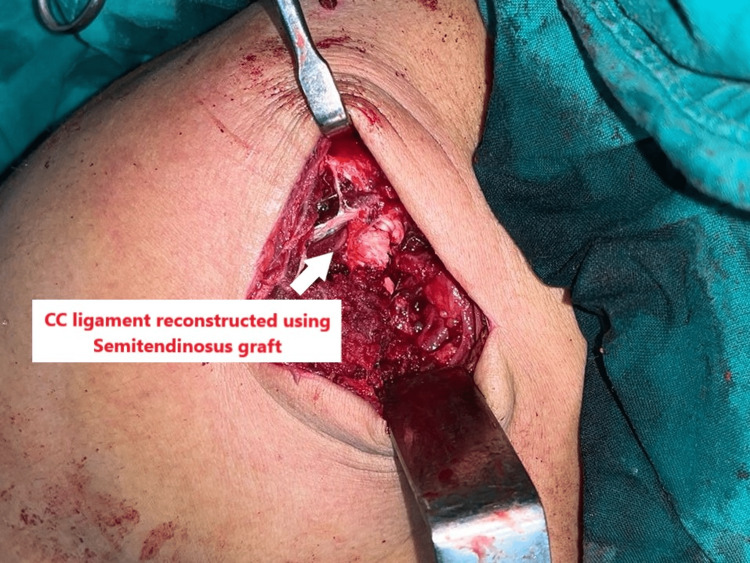
Reconstruction of the coracoclavicular ligament using semitendinosus.

A long-curved hemostat was used for blunt dissection to create windows on either side of the coracoid. Drill holes of 1.8 mm were then constructed in the conoid and trapezoid ligaments' anatomical insertions in the clavicle pointing towards the coracoid. The semitendinosus graft was hooked around the coracoid and delivered through the respective holes. The ends were then tied together with ethibond and vicryl sutures after pulling the medial fragment of the clavicle towards the coracoid held by the tensile strength of the graft, as shown in Figure 7.

The reduction of lateral end clavicle fracture was checked under image intensifier, and closure was done in layers.

Post-operative period

**Figure 8:**
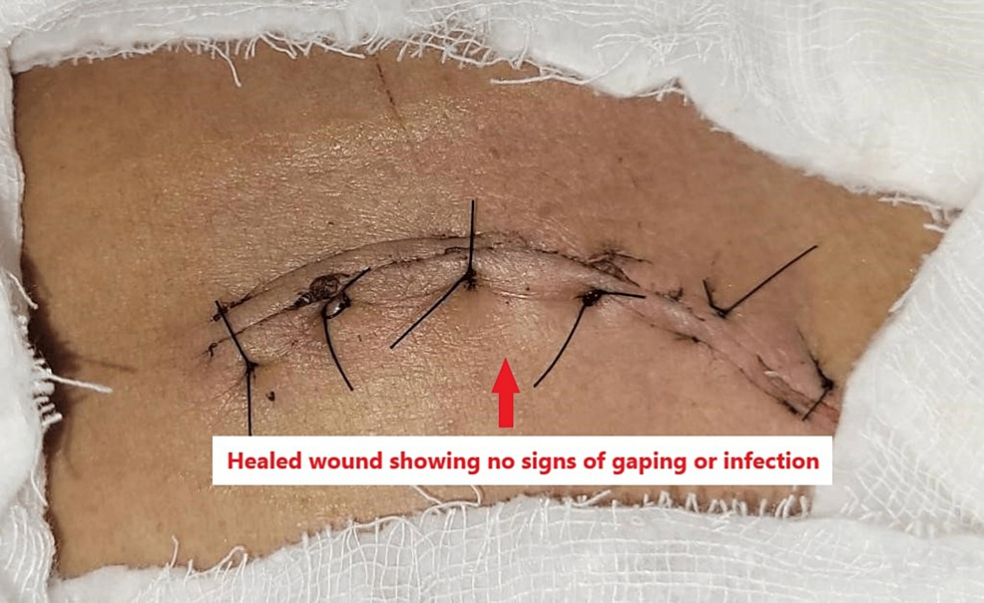
Surgical wound condition on post-operative day 8.

A universal shoulder immobilizer was applied and advised for six weeks to avoid early graft failure. Routine antibiotics and pain medications were prescribed along with dressings on post-operative days 2, 8 and 12 (suture removal), as shown in Figure 8.

**Figure 9:**
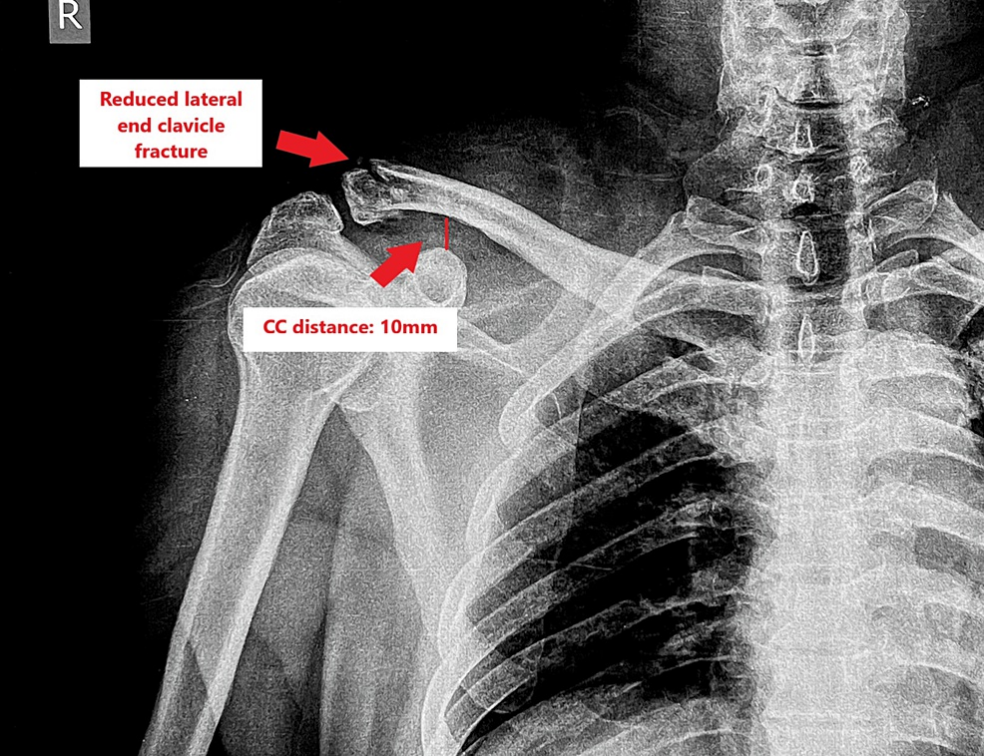
Post-operative radiograph showing anteroposterior (AP) view of the right shoulder.

Shoulder pendular exercises were started as per tolerance. A post-operative radiograph was taken, as shown in Figure 9, which revealed an acceptable reduction of the fracture with a decrease in coracoclavicular distance compared to the pre-operative radiograph.

## Discussion

In type II fractures of the lateral end of the clavicle, there are strong displacement pressures that operate in both the horizontal and vertical directions at the fracture site. These forces comprise the arm's weight, the scapular rotation, the pull of the latissimus dorsi and sternocleidomastoid muscles, as well as the draw of both pectoralis muscles. Therefore, the cornerstone of therapy for these unstable lateral end clavicle fractures has been surgical intervention. High rates of non-union are typically associated with conservative treatment of such fractures. As type IIC fractures according to Cho's classification involve a tiny "extralateral" fragment, hardware options such as locking plates are not recommended.

In our case report, a 60-year-old male with a type IIC lateral end clavicle fracture with coracoclavicular ligament tear underwent ligament reconstruction via a minimal approach without the use of traditional hardware. The main challenge faced during surgery was identifying and harvesting a semitendinosus graft of appropriate length. Post-operative immobilisation with a universal shoulder immobilizer was advised as a precautionary measure to avoid early graft failure. The wound healed optimally without any discharge or signs of infection throughout the post-operative period. Overall, the patient responded well to treatment, and the post-operative period was uneventful.

## Conclusions

The coracoclavicular ligament can be called complex as it comprises the conoid and trapezoid ligaments. The conoid and trapezoid ligaments are continuous inferiorly at the coracoid process attachment but separate at an angle before attaching to the inferior aspect of the clavicle superiorly. It connects the coracoid process of the scapula to the clavicle, and its two-part design prevents the scapula from moving vertically in relation to the clavicle and enables appropriate apposition of the acromion and clavicle.

Cho's Type IIC lateral end clavicle fractures with conoid and trapezoid ligament tears are unstable and require surgical management. Autogenous grafts, such as the semitendinosus, can be used to reconstruct the coracoclavicular ligament to counteract vertical stress forces acting at the fracture site, resulting in a good radiological and clinical outcome with minimal complications.
